# Hope for vascular cognitive impairment: Ac-YVAD-cmk as a novel treatment against white matter rarefaction

**DOI:** 10.1371/journal.pone.0299703

**Published:** 2024-04-17

**Authors:** Yun-An Lim, Li Si Tan, Wei Thye Lee, Wei Liang Sim, Yang Lv, Maki Takakuni, Satoshi Saito, Masafumi Ihara, Thiruma Valavan Arumugam, Christopher Chen, Fred Wai-Shiu Wong, Gavin Stewart Dawe

**Affiliations:** 1 Department of Pharmacology, Yong Loo Lin School of Medicine, National University of Singapore, Singapore, Singapore; 2 Department of Physiology, National University of Singapore, Singapore, Singapore; 3 Department of Neurology, Graduate School of Medicine, Kyoto University, Kyoto, Japan; 4 Department of Neurology, National Cerebral and Cardiovascular Center, Suita, Osaka, Japan; 5 Japan Society for the Promotion of Science, Chiyoda-ku, Tokyo, Japan; 6 School of Life Sciences, La Trobe University, Bundoora, Victoria, Australia; University College London, UNITED KINGDOM

## Abstract

Vascular cognitive impairment (VCI) is the second leading cause of dementia with limited treatment options, characterised by cerebral hypoperfusion-induced white matter rarefaction (WMR). Subcortical VCI is the most common form of VCI, but the underlying reasons for region susceptibility remain elusive. Recent studies employing the bilateral cortical artery stenosis (BCAS) method demonstrate that various inflammasomes regulate white matter injury and blood-brain barrier dysfunction but whether caspase-1 inhibition will be beneficial remains unclear. To address this, we performed BCAS on C57/BL6 mice to study the effects of Ac-YVAD-cmk, a caspase-1 inhibitor, on the subcortical and cortical regions. Cerebral blood flow (CBF), WMR, neuroinflammation and the expression of tight junction-related proteins associated with blood-brain barrier integrity were assessed 15 days post BCAS. We observed that Ac-YVAD-cmk restored CBF, attenuated BCAS-induced WMR and restored subcortical myelin expression. Within the subcortical region, BCAS activated the NLRP3/caspase-1/interleukin-1beta axis only within the subcortical region, which was attenuated by Ac-YVAD-cmk. Although we observed that BCAS induced significant increases in VCAM-1 expression in both brain regions that were attenuated with Ac-YVAD-cmk, only ZO-1 and occludin were observed to be significantly altered in the subcortical region. Here we show that caspase-1 may contribute to subcortical regional susceptibility in a mouse model of VCI. In addition, our results support further investigations into the potential of Ac-YVAD-cmk as a novel treatment strategy against subcortical VCI and other conditions exhibiting cerebral hypoperfusion-induced WMR.

## Introduction

Vascular dementia (VaD) is the second leading cause of dementia with limited treatment options [1, 2]. VaD is preceded by vascular cognitive impairment (VCI) [3], and is characterised by small blood vessel pathology, a decrease in cerebral blood flow (CBF) leading to the development of progressive white matter rarefaction (WMR) [4]. Subcortical VCI is the most common form of VCI [5], but the underlying reasons for subcortical region susceptibility remain elusive.

White matter is enriched in the subcortical brain region [6], but the conditions underlying why cerebral hypoperfusion affects the white matter predominantly in the subcortical region over the cortical region remains unresolved. White matter comprises approximately half of the total brain volume [7] and is the connectivity “highway” responsible for signal transmission between neurons. White matter is predominantly made up of nerve fibres that are wrapped with myelin, which acts as an insulating layer to enable rapid transmission of information within the brain [8]. Indeed, the major constituent of white matter is myelin, which is produced by oligodendrocytes, and is essential for network connectivity linking different brain regions [6, 8, 9]. Myelin loss leads to the formation of WMR, which then develops into a subsequent loss in brain network connectivity, with the latter presenting as white matter hyperintensities in magnetic resonance images (MRI) [10].

Indeed, neuroimaging studies have consistently demonstrated significant associations between chronic cerebral hypoperfusion and the presence of WMR during ageing, VCI and even in other dementias such as AD [11–13]. In non-demented individuals, the presence of white matter hyperintensities is significantly associated with a 2-fold increase in the risk of dementia [14], and white matter hyperintensity burden is significantly correlated with a decline in cognitive performance [15, 16]. Importantly, imaging studies show that white matter pathology precedes cognitive impairment [17–19], suggesting that white matter dysfunction may play an important role in the development of cognitive impairment. If so, elucidating the relationship between CBF and other early major events surrounding the initiation of white matter dysfunction may assist in the development of novel disease-modifying treatments for VCI, which remains limited. Interestingly, recent studies demonstrate significant associations between vascular inflammation and white matter hyperintensities [20, 21], suggesting that the neuro-immune interaction may play a critical role in the etiology VCI/VaD [22–24], but the underlying mechanisms requires elucidation [25, 26].

The bilateral carotid artery stenosis (BCAS) method was established almost two decades ago to study the effects of chronic cerebral hypoperfusion on white matter integrity *in vivo* [27]. Since then, the BCAS method has been used to study the effects of cerebral hypoperfusion on the brain and validated by many research groups worldwide [27–40]. The BCAS model reliably induces cerebral hypoperfusion-induced white matter injury, especially in the subcortical brain region [27, 30], by disrupting the blood-brain barrier (BBB) [37, 41–43]. Importantly, the BCAS mouse model has reliably recapitulated observations in VCI patients [1, 2, 44], supporting its clinical relevance. Briefly, BCAS is performed under anaesthesia and achieved by the bilateral implantation of external micro-coils onto the carotid arteries to induce stenosis, which consistently leads to an approximately 30% decrease in cerebral blood flow (CBF) [27]. The prolonged decrease in CBF results in white matter rarefaction, typically starting from two weeks post BCAS [27–40].

Recent BCAS studies confirmed that inflammasomes play prominent roles in the development of WMR. The process of inflammation is modulated by the activation of inflammasomes [23, 45]. At least four types of inflammasomes have been described [25], which includes the NOD-like receptor family pyrin domain containing 3 (NLRP3) inflammasome [26, 46], which has been shown to be activated by BCAS [47]. Mechanistically, inflammasomal activation leads to the production of active caspase-1 [48], resulting in the proteolytic activation of the proinflammatory interleukin 1-beta (il-1β) from its precursor [49]. Previously, caspase-1 inhibition protects against cerebral demyelination in a mouse model of multiple sclerosis [50], suggesting that caspase-1 may play a role in the regulation of white matter integrity. However, whether caspase-1 plays a role in region susceptibility during cerebral hypoperfusion-associated WMR remains unresolved. Furthermore, given that caspase-1 is the central mediator for inflammasome activation [49], targeting caspase-1 should therefore be beneficial against BCAS-induced white matter injury, but this remains to be confirmed.

Previously, BCAS studies have also demonstrated that BCAS deregulate the BBB permeability via targeting tight junctions [37, 41, 42], which are intercellular adhesions that exist between adjacent endothelial cells lining the lumen of blood vessels [51, 52] that control vascular permeability within the BBB [53]. Examples of tight junction-related proteins include but are not limited to vascular cell adhesion molecule 1 (VCAM-1), occludin, zonula occludens-1 (ZO-1) and claudin-5 [54]. These tight junction proteins have previously been observed to be altered in dementia patients with VCI and Alzheimer’s disease [55–59]. In particular, occludin [41], zonula occludens-1 (ZO-1) [42] and claudin-5 [37, 40] have been shown to be downregulated by BCAS, but the underlying mechanisms remain unresolved. Since inflammation is a known regulator of BBB permeability [60] and inflammation itself is regulated by inflammasomes, it remains to be determined whether these tight junction proteins may be modulated by caspase-1.

Therefore, the overall aim of this study is to assess the relationship between cerebral hypoperfusion and caspase-1 using the BCAS method in mice. The corresponding objective is to assess the putative protective effects of a caspase-1 specific inhibitor, Ac-YVAD-cmk on BCAS-operated mice to study treatment effect on WMR, neuroinflammation and the expression of tight junction proteins within the cortical and subcortical brain regions. We hypothesise that caspase-1 inhibition will ameliorate BCAS-induced WMR and neuroinflammation. We further hypothesise that caspase-1 inhibition will restore the expression levels of BCAS-deregulated tight junction proteins. Finally, we hypothesise that BCAS will exert more deregulation in the subcortical region than in the cortical region.

## Materials and methods

This section will outline the study methodology used for this study. Briefly, we employed the use of a well-established BCAS surgery protocol to induce cerebral hypoperfusion in mice [27–40]. After 15 days post BCAS, brain tissue was obtained and analysed using western blotting and immunohistochemistry. Details are described under each subheading within this section.

### Ethical statement

All in vivo procedures were approved by the National University of Singapore Institutional Animal Care and Use Committee (protocol R17-1235). Experiments were performed in accordance with guidelines for animal experimentation by the National Advisory Committee for Laboratory Animal Research in Singapore and reported according to the ARRIVE guidelines.

### Animals

12–14 weeks old C57BL/6NTac wildtype male mice (each weighing between 25 to 35g) were purchased from InVivos Pte Ltd (Singapore). Mice were assigned to the following experimental groups via block randomisation–Sham, BCAS+ vehicle treatment (10% DMSO in saline) and BCAS+ 10mg/kg Ac-YVAD-cmk. A total of 25 mice were used in this study (N = 7 Sham, N = 9 BCAS+vehicle, N = 9 10mg/kg BCAS+Ac-YVAD-cmk treatment). Animal numbers for each study were determined based on power analysis on a previous pilot study and are comparable to previous BCAS studies [27, 41], and are described in their respective figure legends and accompanying text. Mice were housed in groups of up to 5 mice/cage in different racks within the same room with *ad libitum* access to food and water. Mice were maintained under standard laboratory conditions with an automatic 12-hour light-dark cycle (lights on at 07:00 h).

### Bilateral common carotid artery stenosis (BCAS) surgery and drug administration

To induce cerebral hypoperfusion, we performed well-established BCAS surgery protocol that has been validated by many independent groups [27–38]. Mice were anesthetised with isoflurane before a midline incision was made to expose both common carotid arteries (CCA) just below the carotid bifurcation. The right CCA was carefully exposed from its carotid sheath and sterile silk sutures were then placed underneath the CCA to gently lift and rotate it around a sterile gold-plated stainless steel micro-coil (inner diameter 0.18mm, length 2.5mm; Wuxi Samini Spring Co. Ltd.). The same was then performed for the left CCA before closing the surgical site. Mice in the sham group underwent the same procedure without micro-coil implantation. Body temperature of mice was maintained at 37 ± 0.5°C throughout the surgery. All operated mice were kept under close observation in a warm recovery cage until they regained full consciousness and could freely access food and water before they were placed back into their respective home cages in the animal facility. Mice were further monitored daily for signs of discomfort, weight loss and lethargy until tissue collection. All efforts were made to minimise suffering and were performed within the animal facility during the light cycle.

All treatments were prepared under sterile conditions, aliquoted into autoclaved sterile tubes and kept at -80°C until required. Ac-YVAD-cmk treatment was prepared by dissolving Ac-YVAD-cmk (Cayman Chemical, USA) in sterile dimethyl sulfoxide (DMSO) according to the manufacturer’s recommendations. A fresh aliquot was thawed for each treatment and diluted 1:10 in sterile saline to a final concentration of 10% DMSO (v/v) just before use. Similarly, the vehicle treatment was prepared by diluting sterile DMSO in sterile saline to a final concentration of 10% DMSO (v/v) in saline just before use. A final concentration of 10% DMSO (v/v) is in line with previous studies that assessed the neuroprotective effects of Ac-YVAD-cmk [61, 62]. Treatments were administered intraperitoneally into each mouse immediately after BCAS surgery followed by every alternate day until tissue collection and was determined in a prior pilot study to be beneficial. A total of 7 injections were administered to each BCAS-operated mouse using 30-gauge insulin syringes before tissue collection.

### Cerebral blood flow (CBF) measurement and analysis

A high-resolution laser speckle contrast imager (PeriCam PSI HR system, Perimed Inc.) was used to record baseline CBF before BCAS (Before), immediately after BCAS surgery (After) and just before tissue collection (Final). Body temperature of mice was maintained at 37 ± 0.5°C throughout CBF imaging. Mice were anaesthetised via isoflurane inhalation and placed in the prone position. Hair overlying the skull was removed before a 5–10mm midline sagittal skin incision was made to expose the top of the skull and kept moist with lubricant eye drops (GenTeal, Alcon) for imaging. CBF images were taken using the PSI HR system with a 70-mW built-in laser diode for illumination. A 1388 × 1038 pixels CCD camera fixed at a working distance 10 cm above the skull (speed 19 Hz, and exposure time 6 ms) was used to acquire CBF images. The PIMSoft program (Perimed Inc.) was used to analyse the CBF images. A 15mm^2^ region of interest encompassing the bregma sagittal suture and lambda of the skull was used to determine CBF reading for each image.

### Tissue collection and processing

Fifteen days after BCAS surgery, mice were anaesthetised via isoflurane inhalation for final measurement of CBF. Mice were then euthanised with a 225mg/kg ketamine + 3mg/kg xylazine cocktail injected intraperitoneally before transcardial perfusion with cold 1x phosphate-buffered saline (PBS; Lonza, #17-517Q). One animal from each group were excluded for subsequent analyses due to incomplete perfusion with PBS. The final number of animals used for western blotting and immunohistochemistry analyses were N = 6 Sham, N = 8 BCAS+vehicle, N = 8 10mg/kg BCAS+Ac-YVAD-cmk treatment), and remained comparable with previous BCAS studies [27, 41]. Brains were removed, and cerebral hemispheres separated. The left hemisphere was sub-dissected into cortical and deep subcortical regions containing the basal ganglia, thalamus, and hypothalamus before snap freezing in liquid nitrogen and kept at -80°C until use. The right hemisphere was fixed in 4% paraformaldehyde (PFA) overnight at 4°C before tissue processing was performed.

### Tissue processing, sectioning, and immunohistochemistry

PFA-fixed brain tissue was processed, embedded in paraffin and subsequently sectioned coronally into 5 μm thick sections using a rotary microtome. Sections were mounted on SuperFrost Plus^™^ glass slides (Thermo Scientific, Menzel-Glaser, #J1800AMNZ). Slides were air-dried overnight at room temperature and heated in a dry oven at 60°C for 3 hours for the tissue to adhere strongly to the glass slides in preparation for immunohistochemistry. Sections were deparaffinised in xylene, sequentially rehydrated before heat antigen retrieval was performed using 10mM sodium citrate antigen retrieval buffer (pH 6.0). Then, sections were permeabilised using 1 x PBS containing 1% Triton-X for 10 minutes before incubating with blocking buffer consisting of 3% (v/v) goat serum and 2% (v/v) BSA in 1 x PBS for 1 hour at room temperature. Slides were then incubated with ionised calcium binding adaptor molecule 1 (Iba1) antibody (Abcam, ab178847) overnight at 4°C. The next day, slides were rinsed three times with 1 x PBS before incubating with Alexa Fluor^®^-488 conjugated goat-anti-rabbit secondary antibody (Invitrogen, cat # A32731) for 1 hour at room temperature. Slides were then washed three times with 1 x PBS before mounting in Immu-Mount^™^ and left to cure in the dark for 2 days before imaging was performed. Fluorescence images were taken using the same exposure settings for all sections under 20X magnification using the Leica DFC7000 GT camera mounted on the Leica DM6 B microscope (Leica, Heidelberg, Germany). Images were analysed using ImageJ software (NIH, Bethesda, MD, USA). At least three non-overlapping fields for each region of interest (thalamus, putamen and cortex) were analysed separately by two independent investigators blinded to the experimental groups. The results were averaged to achieve the final intensity and % area readings.

### Luxol fast blue (LFB) staining and scoring

To assess white matter integrity, myelin was stained using the LFB stain kit (Abcam, #ab150675) according to the manufacturer’s protocols. Briefly, after deparaffinisation, four sections from each mouse brain were stained with LFB solution overnight at 56°C. Excess staining was removed with 95% ethanol and rinsed in deionised water. Sections were differentiated with 0.05% lithium carbonate and 70% ethanol solutions before counterstaining with cresyl echt violet. Sections were then dehydrated stepwise, cleared in xylene before mounting with anhydrous Neo-Mount^®^ (Merck, 109016). Bright field images were taken at 20X magnification using the Leica DFC7000 GT camera mounted on the Leica DM6 B microscope (Leica, Heidelberg, Germany). White matter injury was evaluated at the paramedial corpus callosum. The degree of white matter changes was determined using the following scoring: normal (Grade 0), disarrangement of nerve fibres (Grade 1), formation of marked vacuoles (Grade 2), and loss of myelinated fibres (Grade 3), as previously performed [23]. Two sections from each brain were analysed separately by two independent investigators blinded to the experimental groups. The results were averaged to achieve the final LFB scores.

### Western blotting

Snap-frozen brain tissue stored at -80°C was thawed on ice and extracted with ice-cold Tissue Protein Extraction Reagent (T-PER^™^, Thermo Fisher Scientific, #78510) supplemented with 50mg/ml PhosSTOP phosphatase inhibitor tablets and 50mg/ml cOmplete^™^ EDTA-free Protease Inhibitor Cocktail tablets (Roche, #4906837001 and #4693159001, respectively). Each tissue sample was sonicated briefly on ice (30% amplitude, Vibra-cell CV188 sonicator) until a homogeneous suspension was obtained. Samples were then incubated on ice for 10 minutes before centrifugation was performed at 21,100 x g for 15 minutes at 4°C. The supernatant was retrieved and centrifuged for a second time to remove any remaining debris. The final supernatant was aliquoted and stored at -80°C until use. Protein concentrations were determined using the DC-Protein assay (Bio-Rad, #500–0116).

Equal amounts of proteins were separated on 8%–15% Tris-Glycine-SDS gels and transferred onto nitrocellulose membranes (0.2 μM pore size, Bio-Rad, #1620112). Membranes were blocked with 5% BSA (HyClone, #SH30574.02) or 5% non-fat milk (Bio-Rad, #1706404) in Tris-Buffered Saline containing 0.1% Tween 20 (TBST) at room temperature for 1 hour, before primary antibody incubation (1:1000) in 5% blocking buffer (BSA or non-fat milk) in TBST at 4°C overnight with gentle agitation. The following primary antibodies were used in this study: myelin basic protein (Cell Signalling Technology, #78896), OLIG2 (Santa Cruz, sc-515947), NLRP3 (Adipogen, AG208-0014-C100), cleaved caspase-1 (Cell Signalling Technology, #67314), cleaved caspase 11 (Abcam, ab22684), IL-1β (Cell Signalling Technology, #12426S), VCAM-1 (Santa Cruz, sc-8304), zonula occludens-1 (ZO-1) (Santa Cruz Biotechnology, #sc-33725), occludin (Santa Cruz Biotechnology, #sc-133256) and claudin-5 (Santa Cruz Biotechnology, #sc-374221). β-Actin (Sigma-Aldrich, #A2228) was used as the loading control for densitometry analysis. Blots were then washed 3 x 10 minutes with TBST before HRP-conjugated secondary antibody incubation for 1 hour at room temperature. The following secondary antibodies were used in this study: anti-mouse IgG (Sigma-Aldrich, #A4416), anti-rabbit IgG (Sigma-Aldrich, #A0545), or anti-rat IgG (Santa Cruz Biotechnology, #sc-2006) in 1% blocking buffer. Blots were washed 3 x 10 minutes with TBST and visualised using the Clarity Western ECL Substrate or Clarity Max Western ECL Substrate (Bio-Rad, #1705061 and #1705062) in a Chemidoc^™^ XRS imager machine. Images were analysed using ImageJ software (National Institutes of Health, Bethesda, Maryland, USA) to obtain densitometry readings.

### Statistical analysis

Data are expressed as means ± SEM. Data were analysed by one-way ANOVA with a Bonferroni correction using SPSS Statistics version 22.0 (IBM Corp, Armonk, NY, USA). A p-value less than 0.05 is considered statistically significant.

## Results

### Ac-YVAD-cmk ameliorates BCAS-induced CBF reduction

**Fig 1:**
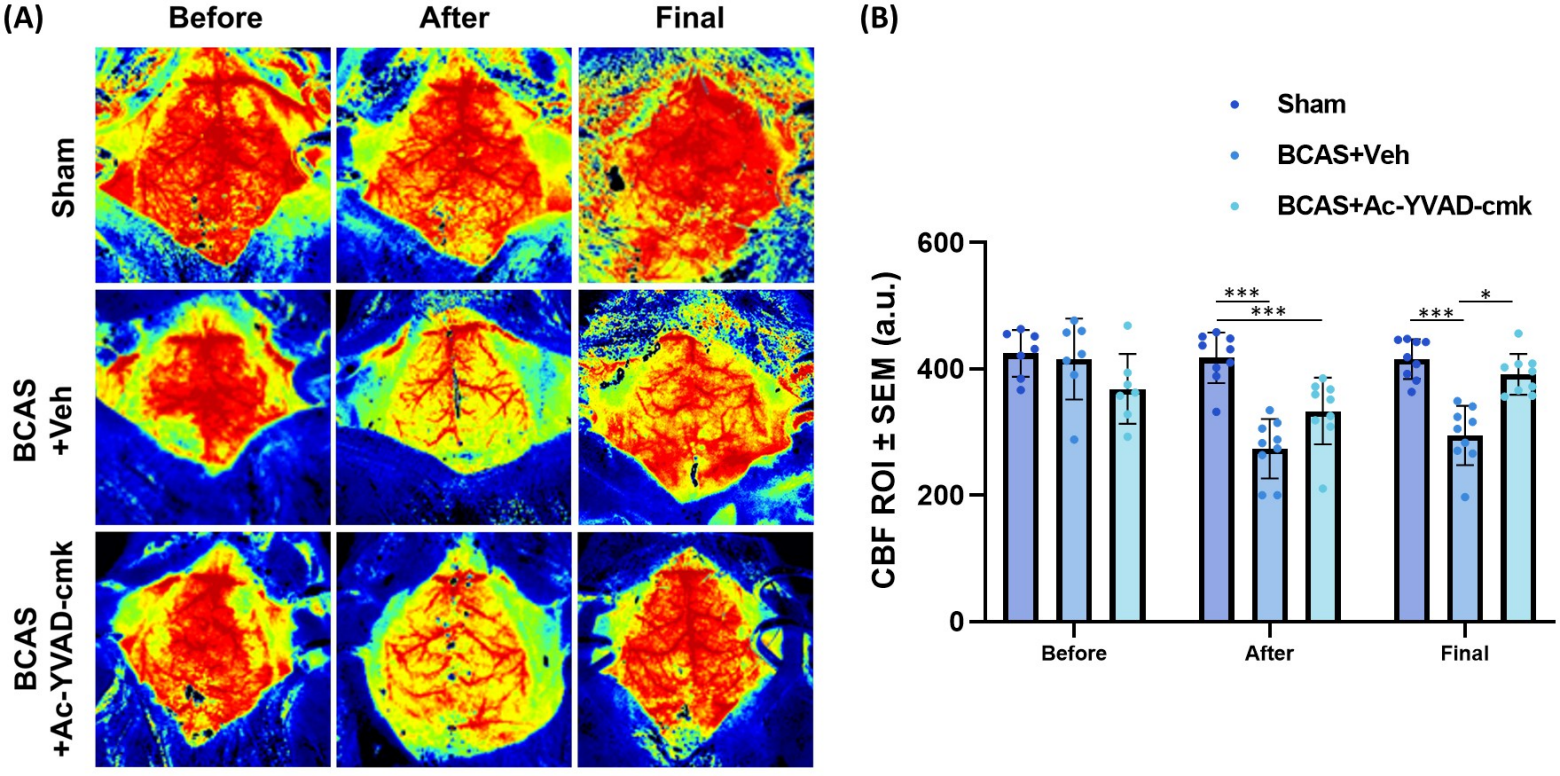
Cerebral blood flow (CBF) measurements. (A) Representative laser speckle flowmetry CBF images across different time points. (B) BCAS induced a significant decrease in CBF, which was significantly restored by treatment with Ac-YVAD-cmk (N = 7 to 9 per group, one-way ANOVA with Bonferroni correction, *p<0.05; ***p<0.001, vs Sham group.

To confirm that BCAS was performed successfully in this study, CBF readings for each mouse were obtained at three time points, corresponding to (i) immediately before BCAS (Before), (ii) immediately after BCAS (After) and (iii) immediately before tissue collection (Final). Representative CBF images are presented in Fig 1A. Analysis of CBF measurements show that BCAS consistently induced an approximate 30% decrease in CBF (Before vs After), in line with previously published findings [27, 31]. Interestingly, treatment with Ac-YVAD-cmk induced a small but significantly increase in CBF when compared to BCAS+Veh group, and the CBF of the Ac-YVAD-cmk group was no longer significantly different from the Sham group (Fig 1B).

### Ac-YVAD-cmk restores white matter integrity in BCAS-operated mice

**Fig 2:**
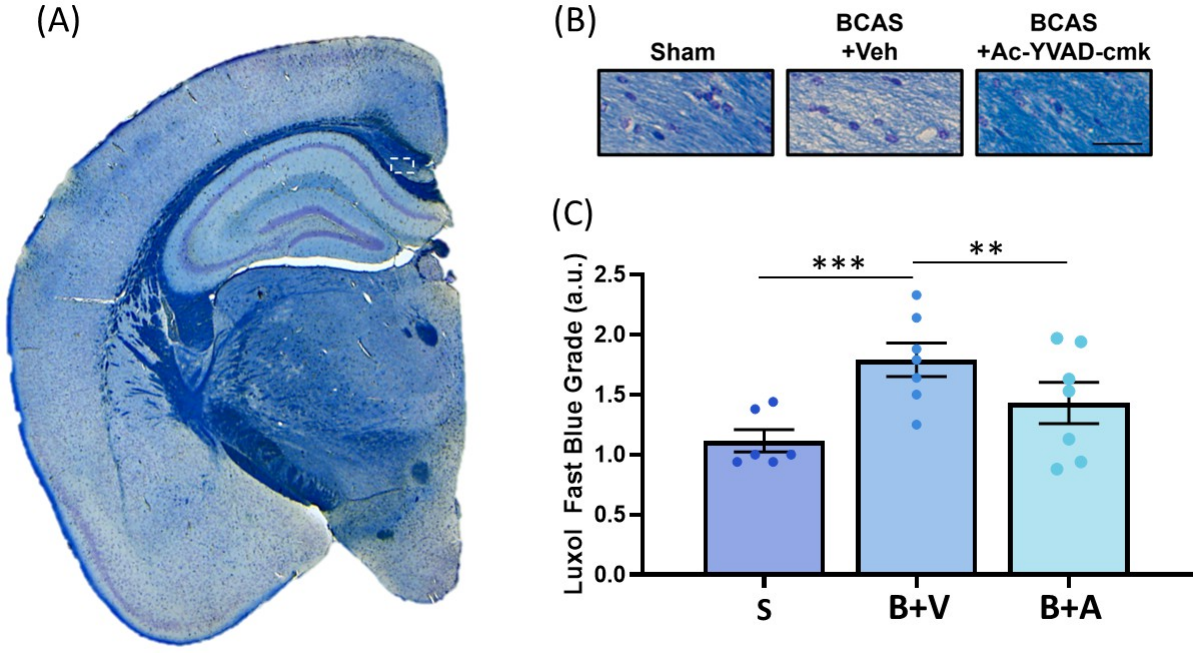
Effect of Ac-YVAD-cmk on BCAS-induced white matter lesions. (I) Location of the corpus callosum used to quantify BCAS-induced white matter loss (white dotted rectangle). (II) Representative figures of Luxol Fast Blue (LFB) staining at 20X, scale bar = 20μm. (III) Statistical analysis shows that the LFB score was significantly increased in the BCAS + Veh group when compared to the Sham, while treatment with Ac-YVAD-cmk significantly restored LFB scoring comparable to Sham group (N = 6 to 7 per group, one-way ANOVA with Bonferroni correction, **p<0.01; ***p<0.001).

Next, we sought to determine the effects of Ac-YVAD-cmk on white matter integrity. To assess the degree of myelination at the corpus callosum, we stained paraffin-embedded brain sections with Luxol fast blue (LFB) stain solution, as we performed previously [38]. LFB scores range from 0 to 3, and a higher LFB score means a higher extent of demyelination as previously described [63]. Since BCAS-induced hypoperfusion is known to induce white matter after 14 days [27], if Ac-YVAD-cmk is able to significantly restore CBF, then Ac-YVAD-cmk should ameliorate BCAS-induced white matter injury in our study. Fig 2A shows the location of the corpus callosum that was used to quantify myelination. Compared to the Sham group, BCAS induced a loss of myelin fibres and increased vacuolisation (Fig 2B), corresponding to a significant increase in the LFB score (Fig 2C), confirming previous studies [32, 35]. Importantly, we observed that treatment with Ac-YVAD-cmk maintained myelin integrity (Fig 2B) and LFB score remained comparable to the Sham group (Fig 2C).

### Ac-YVAD-cmk restores myelin levels and attenuates neuroinflammation in the subcortical region of BCAS-operated mice

**Fig 3:**
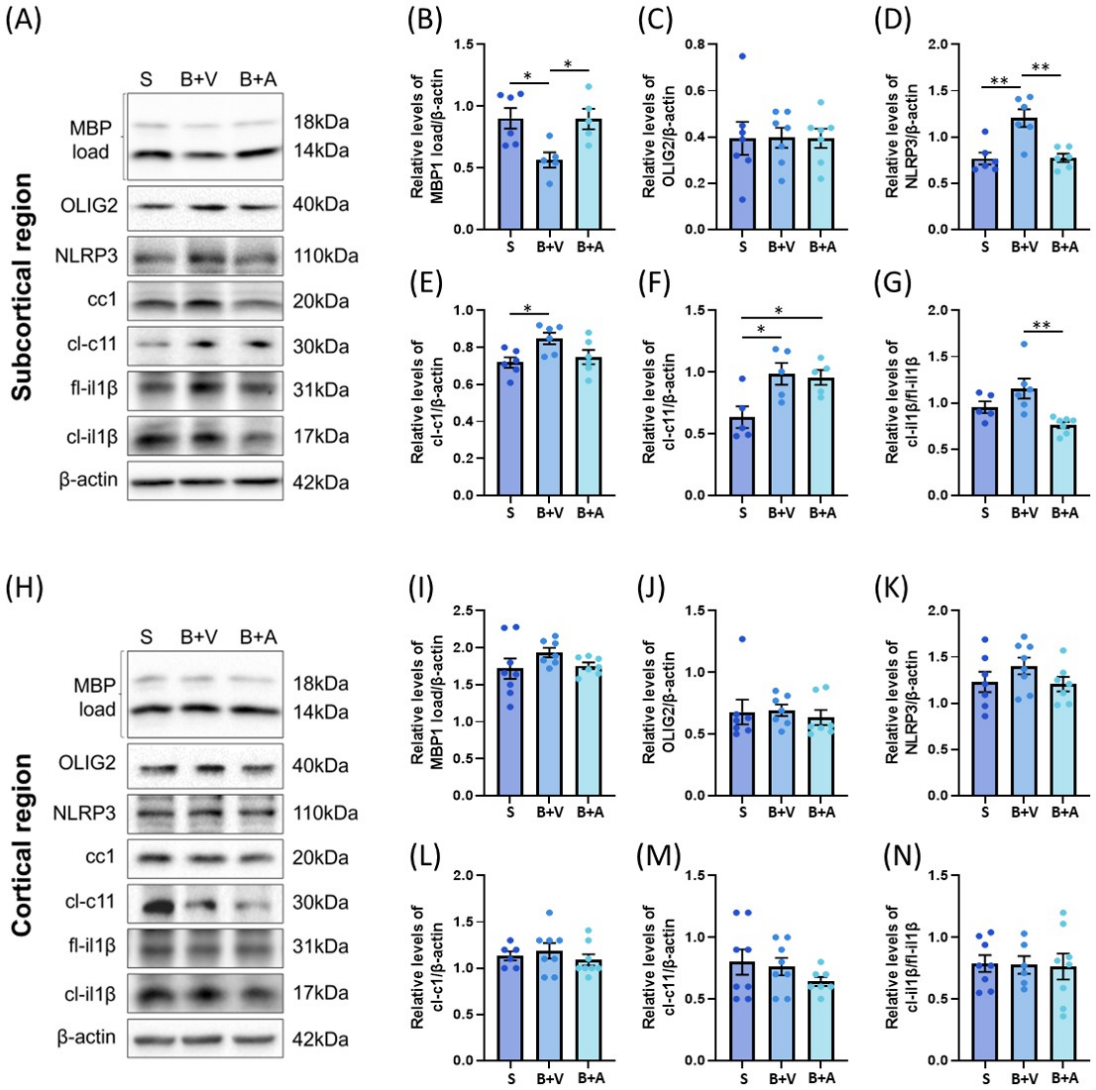
Effect of BCAS on myelin and inflammation in subcortical and cortical tissue. (A-G) Analysis of the subcortical region. (A) Representative immunoblots of subcortical region 15D post BCAS (S = Sham, B+V = BCAS+Veh, B+A = BCAS+Ac-YVAD-cmk). (B to G) Densitometry analysis of immunoblots. (B) BCAS induced a significant decrease in levels of myelin basic protein (MBP) load, which was significantly restored with Ac-YVAD-cmk treatment. (C) BCAS had no effect on the levels of OLIG2. (D) BCAS significantly increased the levels of NLRP3, which was significantly restored with Ac-YVAD-cmk treatment. (E) BCAS induced a significant increase in levels of cleaved caspase-1 (cl-c1), which was no longer significantly different to Sham group with Ac-YVAD-cmk treatment. (F) BCAS induced a significant increase in levels of cleaved caspase-11 (cl-c11), which remained elevated despite treatment with Ac-YVAD-cmk. (G) The level of cleaved interleukin-1beta (cl-il1β)/full length interleukin-1beta (fl-il1β) ratio trended higher in the BCAS+Veh group when compared to the Sham group but did not reach significance. However, treatment with Ac-YVAD-cmk resulted in a significant decrease in the cl-il1β/fl-il1β ratio when compared to the BCAS+Veh group. (H-N) Analysis of the cortical region. (H) Representative immunoblots of cortex region 15D post BCAS (S = Sham, B+V = BCAS+Veh, B+A = BCAS+Ac-YVAD-cmk). (I to N) Densitometry analysis of immunoblots. BCAS and Ac-YVAD-cmk had no significant effect on the levels of (I) MBP load, (J) OLIG2, (K) NLRP3, (L) cl-c1, (M) cl-c11, and (N) cl-il1β/fl-1β ratio (N = 5 to 8 per group), one-way ANOVA with Bonferroni correction, *<p = 0.05; **p<0.01).

To study the effects of Ac-YVAD-cmk on myelin levels and neuroinflammation in the subcortical and cortical brain regions, we performed western blotting, and the results are outlined in Fig 3. Representative blots for the subcortical region are shown in Fig 3A. Analysis showed that BCAS significantly decreased MBP1 expression, which is in line with our previous observations [38, 64]. Importantly, MBP1 expression was completely restored with Ac-YVAD-cmk treatment (Fig 3B). Since MBP1 is an established marker for demyelination [65], our result confirms that Ac-YVAD-cmk is protective against hypoperfusion-induced white matter demyelination. To confirm the action of Ac-YVAD-cmk on oligodendrocytes, we also assessed the levels of OLIG2, as it is an established pan-oligodendrocyte marker [66, 67]. The levels of OLIG2 remained unaltered across all groups (Fig 3C). Together, the results indicate that BCAS induces cerebral demyelination in the absence of oligodendrocyte loss in our study.

Next, we ascertained the effects of Ac-YVAD-cmk on the NLRP3/caspase-1/il-1β pathway by studying the expression levels of these proteins. We observed that BCAS induced a significant increase in NLRP3 expression, which was significantly restored to the level of the Sham group upon treatment with Ac-YVAD-cmk (Fig 3D). Similarly, BCAS significantly increased the expression of cleaved caspase-1 (cl-c1), which became comparable to the Sham group upon Ac-YVAD-cmk treatment (Fig 3E). Although Ac-YVAD-cmk is an irreversible caspase-1 inhibitor [68], it has previously been observed to exert some effect on the activities of caspase-4 and 5 [69]. As the murine orthologue of caspase-4 and 5 is caspase-11 [70], in order to rule out potential unspecificity of Ac-YVAD-cmk in our study model, we assessed the expression level of cleaved caspase-11 (cl-c11) as a readout for its activity (Fig 3F). We observed that BCAS significantly increased the expression of cl-c11, which remained upregulated despite Ac-YVAD-cmk treatment. Next, we assessed the ratio of cleaved interleukin 1beta (cl-il1β)/full length interleukin 1beta (fl-il1β) as a readout for caspase-1 activity. We observed that BCAS induced an increase in the cl-il1β/fl-il1β ratio, which neared significance (Fig 3G). Importantly, treatment with Ac-YVAD-cmk significantly decreased the ratio of cl-il1β/fl-il1β to become comparable to the Sham group (Fig 3G).

In contrast, BCAS did not significantly alter any of the assessed targets in the cortical region. Representative blots for the cortical region are shown in Fig 3H. Specifically, no significant differences were observed in the levels of MBP1 (Fig 3I) and OLIG2 (Fig 3J) across all experimental groups within the cortex. Although the expression levels of NLRP3 (Fig 3K), cl-cl1 (Fig 3L) and cl-il1β/fl-il1β (Fig 3N) trended higher in the BCAS + Veh group when compared to the Sham and BCAS + Ac-YVAD-cmk groups, none of them reached statistical significance. The level of cl-c11 trended lower upon treatment with Ac-YVAD-cmk but remained statistically unaltered (Fig 3M).

### Ac-YVAD-cmk ameliorates BCAS-induced Iba1 immunoreactivity in the subcortical and cortical regions

**Fig 4:**
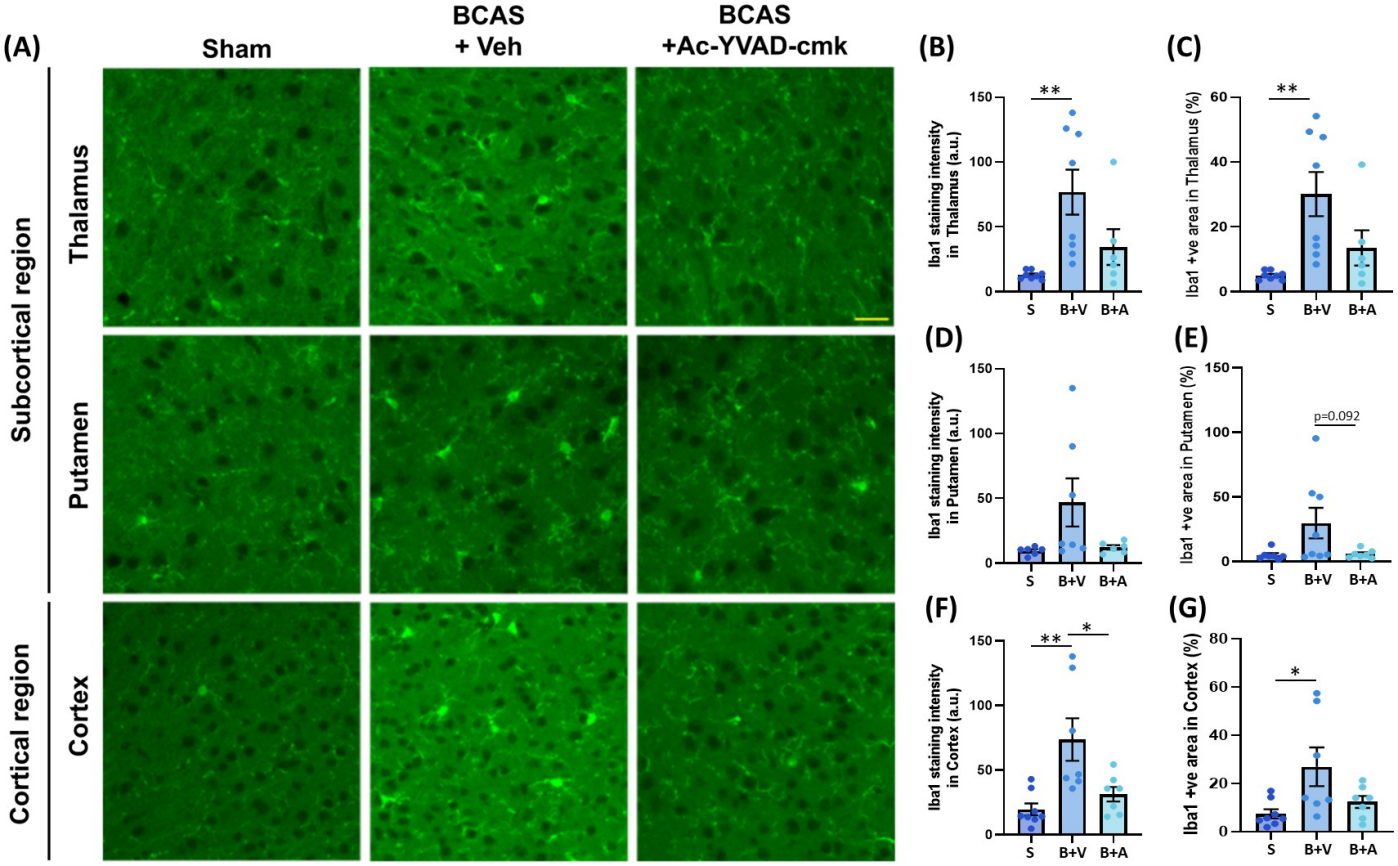
Effect of BCAS on Iba1 immunoreactivity in the subcortical and cortical brain regions. (A) Representative micrograph images showing the effect of BCAS and Ac-YVAD-cmk on the subcortical (thalamus and putamen) and cortical brain regions (Scale bar = 30μm, 20X). (B to G) Immunohistochemical analysis of Iba1 immunoreactivity in the Thalamus, putamen, and the cortex. (B & C) For the thalamus, when compared to the Sham group, Iba1-immunoreactivity intensity as well as Iba1 positive % area was significantly increased in the BCAS+Veh group but were no longer statistically different to the BCAS+Ac-YVAD-cmk group. (D & E) For the putamen, when compared to the Sham group, Iba1-immunoreactivity intensity as well as Iba1 positive % area was increased in the BCAS+Veh group but did not reach significance. Iba1 immunoreactivity and positive % area remained comparable between the Sham and BCAS+Ac-YVAD-cmk groups. (F) For the cortex, when compared to the Sham group, Iba1-immunoreactivity intensity was significantly increased in the BCAS+Veh group, which was significantly reduced in the BCAS+Ac-YVAD-cmk group. (G) The Iba1 positive % area was significantly increased in the BCAS+Veh group when compared to the Sham group. The Iba1 positive % area was comparable between the Sham and BCAS+Ac-YVAD-cmk groups (N = 6 to 8 per group, one-way ANOVA with Bonferroni correction, *<p = 0.05; **p<0.01).

To confirm the regional effects of BCAS on neuroinflammation in the subcortical and cortical brain regions, we stained BCAS-operated mouse brain sections with an Iba1 antibody (Fig 4). Within the thalamus, we observed that BCAS induced a significant increase in Iba1-positive immunoreactivity when compared to the Sham group, which was attenuated by Ac-YVAD-cmk (Fig 4B). Correspondingly, BCAS induced a significant increase in the Ibal1-positive % area when compared to the Sham group, which was also attenuated by Ac-YVAD-cmk (Fig 4C). The same trends were observed in the putamen (Fig 4D & 4E) but did not reach significance. Within the cortex, BCAS induced a significant increase in the intensity level of Iba1, which was significantly reduced with Ac-YVAD-cmk treatment (Fig 4F). Iba1-positive % area was significantly increased in the BCAS+Veh group when compared to the Sham group, which was reduced in the BCAS+Ac-YVAD-cmk group to a level comparable to the Sham group (Fig 4G).

### BCAS significantly decreased the expression of tight junction-related proteins in the subcortical but not in the cortical region

**Fig 5:**
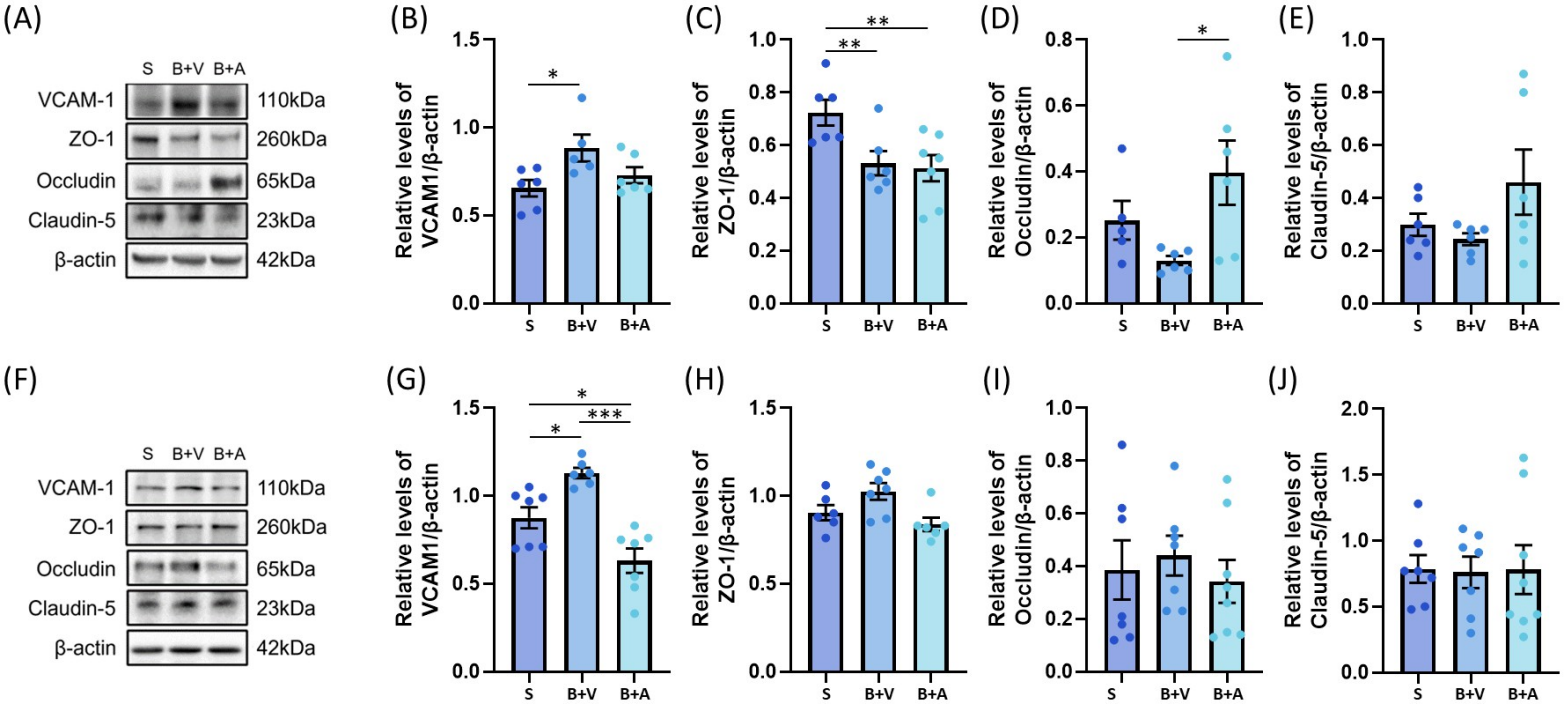
Effect of BCAS on vascular inflammation and tight junction-related proteins in subcortical and cortical tissue. (A) Representative immunoblots of subcortical tissue (S = Sham, B+V = BCAS+Veh, B+A = BCAS+Ac-YVAD-cmk). (B to E) Densitometry analysis of immunoblots of the subcortical tissue. (B) BCAS induced a significantly increase in levels of VCAM-1 when compared to Sham group but became comparable to Sham group after Ac-YVAD-cmk treatment. (C) BCAS significantly decreased the level of subcortical ZO-1 when compared to the Sham group and remained depressed even with Ac-YVAD-cmk treatment. (D) BCAS decreased the level of subcortical occludin when compared to Sham group that neared significance but was significantly increased upon Ac-YVAD-cmk treatment. (E) The subcortical level of claudin-5 was not significantly altered by BCAS or Ac-YVAD-cmk when compared to Sham group. (F) Representative immunoblots of cortical tissue. (G to J) Densitometry analysis of immunoblots of the cortical tissue. (G) BCAS induced a significantly increase in VCAM-1 when compared to the Sham group but became significantly lower than that of Sham group upon treatment with Ac-YVAD-cmk. The levels of ZO-1 (H), occludin (I), and claudin-5 (J) remained significantly unaltered by BCAS or Ac-YVAD-cmk in the cortical region (N = 5 to 8 per group, one-way ANOVA with Bonferroni correction, *<p = 0.05; **p<0.01, ***p<0.001).

To assess if BCAS and Ac-YVAD-cmk may differentially affect the blood brain barriers within the subcortical and cortical regions, western blotting was performed to assess the expression of tight junction-related proteins (Fig 5A & 5F). VCAM-1 expression was significantly upregulated in both the subcortical (Fig 5B) and the cortical (Fig 5G) regions. However, their responses to Ac-YVAD-cmk treatment differed in magnitude. In the subcortical region, Ac-YVAD-cmk treatment restored the level of VCAM-1 to a level comparable to the Sham group (Fig 5B). In the cortical region, Ac-YVAD-cmk treatment led to significant reductions in the expression of VCAM1 when compared to the vehicle-treated and Sham groups (Fig 5G).

Next, western blotting was performed to ascertain the putative effects of Ac-YVAD-cmk on the expression levels of tight junction-related proteins in the subcortical (Fig 5A) and cortical (Fig 5F) regions. In the subcortical region, we observed that BCAS significantly downregulated the expression of ZO-1, which remained depressed despite Ac-YVAD-cmk treatment (Fig 5C). BCAS induced a reduction in occludin that almost reached significance (Fig 5D) that became significantly elevated in the Ac-YVAD-cmk treated group when compared to the vehicle treated group (Fig 5D). The level of claudin-5 remained unchanged across all experimental groups in our study (Fig 5E). In contrast, none of the expression levels of ZO-1 (Fig 5H), occludin (Fig 5I) and claudin-5 (Fig 5J) was altered across all experimental groups within the cortical region.

In this study, we observed that Ac-YVAD-cmk restored CBF (Fig 1), abolished WMR (Fig 2), attenuated myelin loss, NLRP3-caspase-1-il1β axis and neuroinflammation (Figs 3 & 4) and restored the expression levels of tight junction-related proteins (Fig 5) in the BCAS mouse model of VCI. Our study demonstrates that BCAS induced significant effects only within the subcortical region, but not the cortical region, which may contribute to known susceptibility in VCI. Our results also support the role of caspase-1 as an early contributor to the development of hypoperfusion-related white matter degeneration.

## Discussion

According to the World Health Organisation (WHO), there are currently over 55 million dementia patients worldwide [71]. Based on current trends, dementia patient numbers are predicted to exceed 150 million by 2050 [72]. Against the backdrop of a global ageing population and a corresponding rapidly increasing socio-economic burden, there is an urgent impetus to develop efficacious treatments against this debilitating condition, which remains lacking. Subcortical VCI is the most common form of VCI (a prodrome of VaD that is the second most common form of dementia) [3], but the reasons underlying subcortical region susceptibility remain unclear [4, 73]. Previous BCAS studies revealed that deeper brain structures in the subcortical region are more affected than those in the cortex [30, 34] but the possible mechanisms underlying the observed disparity remained unexplored. Our study builds upon previous BCAS studies [30, 35, 41] by assessing the relationship between cerebral hypoperfusion and caspase 1. Our results show that hypoperfusion induces key regional differences between the subcortical and cortical regions in regard to white matter integrity, inflammasomal activation, microglial activation and tight junction protein expressions.

We showed that BCAS induced significant WMR in the corpus callosum after 15 days post BCAS, which is in line with previous studies showing that WMR is typically observed starting from two weeks post BCAS [27–38]. Importantly, we demonstrated that prophylactic treatment with Ac-YVAD-cmk prevented white matter demyelination, supporting the central role of caspase-1 as an early contributor to the hypoperfusion-mediated WMR process. It remains unknown whether Ac-YVAD-cmk treatment is able to promote white matter regeneration after WMR has been established, which may be worthwhile to ascertain in future studies.

In line with this, we showed that treatment with Ac-YVAD-cmk prevented cerebral demyelination in the absence of any overt oligodendrocyte loss within the subcortical region, and is in line with previous studies since oligodendrocyte loss typically only occurs after four weeks post BCAS [28, 29]. In contrast, we found that myelin expression remained largely resistant to the effects of BCAS within the cortex. Our finding confirms the results from a previous study that observed no change in cortical MBP1 levels up to 30 days BCAS post-surgery [35]. The reasons as to why the cortex is preferentially spared remains unclear but might be due to differences in cerebrovascular reserves within the white matter when compared to the grey matter in human brains [74–76]. This difference has also also been observed in mice in a previous study showing lower vessel densities within the white matter when compared to the grey matter [74–77], which may underline subcortical regional susceptibility observed in the current study. Future BCAS studies to compare the effects of caspase-1 inhibition on blood vessel densities, morphologies and angiogenesis capacities within the subcortical and cortical regions will likely yield relevant findings to further understanding into the relationship between CBF and white matter homeostasis for human VCI.

Previous studies showed that inflammation is significantly associated with white matter dysfunction before [78] and during VCI [79], suggesting that inflammation plays an early important role in the etiology of white matter dysfunction [80]. Indeed, the NLRP3 and AIM2 inflammasomes were found to be activated in the brains of BCAS-operated mice [47, 81], but it remained unclear whether modulating the activity of their common mediator caspase-1 may be beneficial. Our study builds upon previous studies and confirmed that the NLRP3-caspase-1 axis is indeed activated during BCAS [82–84]. Importantly, our results demonstrate that the NLRP3-caspase-1 axis was only activated in the subcortical, but not in the cortical region, which may underline previous observations regional susceptibility [4, 35, 73]. These findings indicate that BCAS exerts differential effects on the inflammatory microenvironments of different brain regions, which may be of interest in future follow-up studies. Unexpectedly, we also showed that caspase-1 inhibition regulates NLRP3 expression, which suggests that caspase-1 activity regulates NLRP3 expression and expands our current understanding of the relationship between NLRP3 and caspase-1.

The thalamus and putamen are two major regions within the subcortex that are known to be affected in VCI [85] and AD [86]. Our results indicate that BCAS induced varying extents of microglial activation in these regions, which may affect treatment considerations as they are known to play varying roles in cognition [87, 88]. Aside from this, although the result from our Iba1 immunofluorescence study are in line with a previous study that used CD11b to label microglia [41], it should be noted that these markers are also expressed by other immune cell types such as leukocytes and macrophages [89], which may also contribute to subcortical regional susceptibility. Together, these findings raise the possibility that different immune cell types may contribute to inflammatory response during hypoperfusion and should be explored in future studies.

VCAM-1 is a protein that is induced on endothelial cells during inflammation and is a commonly used marker for vascular inflammation [90]. Our results showed that VCAM1 expression is significantly increased by BCAS in both the subcortical and cortical regions and is caspase-1-dependent. As vascular inflammation is closely related to cerebral hypoperfusion [91], our results suggest that VCAM-1 is a target for caspase-1. It is interesting to note that VCAM-1 expression levels across the experimental groups are generally inversely correlated with their respective final CBF readings in our study, which is in line with previous studies that showed VCAM-1 expression is inversely regulated by shear stress [92–94]. Future follow-up studies may assess whether modulating VCAM-1 expression affects the development of hypoperfusion-induced WMR.

It is known that vascular inflammation is typically observed alongside BBB dysfunction [95]. BBB integrity is regulated by tight junction proteins such as occludin, ZO-1 and claudin-5, which have all been shown to be downregulated by BCAS [37, 40–42]. Our results indicate that BCAS induced significant downregulation in ZO-1 and occludin expressions in the subcortical region, which are in line with previous observations [37, 41, 42]. Importantly, only occludin, but not ZO-1, was found to respond to caspase-1 inhibition. In contrast, claudin-5 expression was not altered in our study and is similar to previous observations [41]. We also show that the expressions of these tight junction proteins are unaltered within the cortical region, suggesting that cortical BBB integrity is likely unaffected by BCAS in our study. Our findings indicate that BCAS induces caspase-1 and caspase-1 independent pathways that target different tight junction proteins and warrants further studies. Our results also suggest that disparities in tight junction protein expressions observed in the subcortical and cortical regions may contribute to regional susceptibility, warranting further investigation.

There are several limitations to our study. Firstly, although we confirmed the specificity of Ac-YVAD-cmk on caspase-1 in our study, we only analysed il-1β in our study. As caspase-1 has multiple downstream targets such as il-18 and gasdermin-D amongst others [96, 97], future studies may build upon this study to ascertain the contributions of these downstream targets at different time points along the process of WMR development. Future studies may also wish to employ caspase-1 knockout mice to validate our findings to confirm the role of caspase-1 in hypoperfusion-induced WMR. Secondly, as our study only focused on the early effects of caspase-1 inhibition on hypoperfusion-induced white matter injury, we were unable to ascertain whether caspase-1 will be beneficial after WMR is established remains unknown. Future studies with longer time points with delayed treatment schedules are needed to address this. Elucidating this will ascertain the role of caspase-1 in myelin regeneration, which has remained unstudied and necessary since white matter dysfunction precedes the onset of cognitive impairment [17–19]. Future studies could build upon ours to assess the effects of caspase-1 inhibition on oligodendrocyte number, grey matter, and memory since they occur at time points later than that assessed in our study [27–29, 36]. Next, we were unable to compare the CBF in different regions of the brain for our study due to machine limitations, so future studies may consider adopting additional imaging methods such as arterial spin labelling magnetic resonance imaging (ASL-MRI) [31] and/or other high resolution deep imaging techniques [98] to further compare CBF alterations within the various brain regions of interest. In line with this, future studies could assess vessel density/morphology as they are affected during cerebral hypoperfusion [99]. Furthermore, as we had administered Ac-YVAD-cmk via i.p. injections, we cannot rule out the possibility that it exerts any systemic/off-target effects that may contribute to our observations in this study, which should be resolved in future studies. Furthermore, although Ac-YVAD-cmk has consistently shown neuroprotective benefits across a variety of studies [61, 62, 68, 100], it remains unclear whether it enters the brain or exerts its benefits only at the level of the BBB, even though cerebral hypoperfusion is known to cause BBB breakdown [101]. Whether Ac-YVAD-cmk enters the brain to exert its neuroprotective effects requires clarification in future studies using different model systems [102], and addressing this should be useful to help translate our findings into the clinical setting.

## Conclusion

It has long been established that inflammation is associated with CBF during normal ageing [103] and across various disease conditions such as acute stroke [104–106] and chronic dementia [91, 107], but its role during VCI remains unresolved. Our findings highlight the possibility of caspase-1 inhibition as a possible novel anti-inflammatory treatment strategy against cerebral hypoperfusion-induced white matter injury, microglial activation, and tight junction protein deregulation. These results support further assessments using different caspase-1 inhibition methodologies to study its role in cerebral hypoperfusion in future studies. Importantly, we demonstrated that cerebral hypoperfusion preferentially affects the subcortical brain region over the cortical brain region via caspase-1-dependent and independent targets, which may contribute towards subcortical regional susceptibility observed in subcortical VCI patients [3, 5, 85], which warrants further investigations. Collectively, the findings arising from this study support further evaluation of caspase-1 inhibition as a potential novel prophylactic treatment strategy against cerebral hypoperfusion-associated white matter injury, which may be especially relevant for patients who suffer from established risk factors of VCI [108].

## Supporting information

S1 Fig Raw images for Fig 3. Western blots for Fig 3.
(TIF)

S2 Fig Raw images for Fig 5. Western blots for Fig 5.
(TIF)

S1 File Raw values for graphs. Raw values for graphs for Figs 1 to 5.
(XLSX)
