# Supplementary figures and images for: Hope for vascular cognitive impairment: Ac-YVAD-cmk as a novel treatment against white matter rarefaction
Source: PLoS One. 2024 Apr 17;19(4):e0299703. doi: 10.1371/journal.pone.0299703 (PMC11023579; doi:10.1371/journal.pone.0299703)

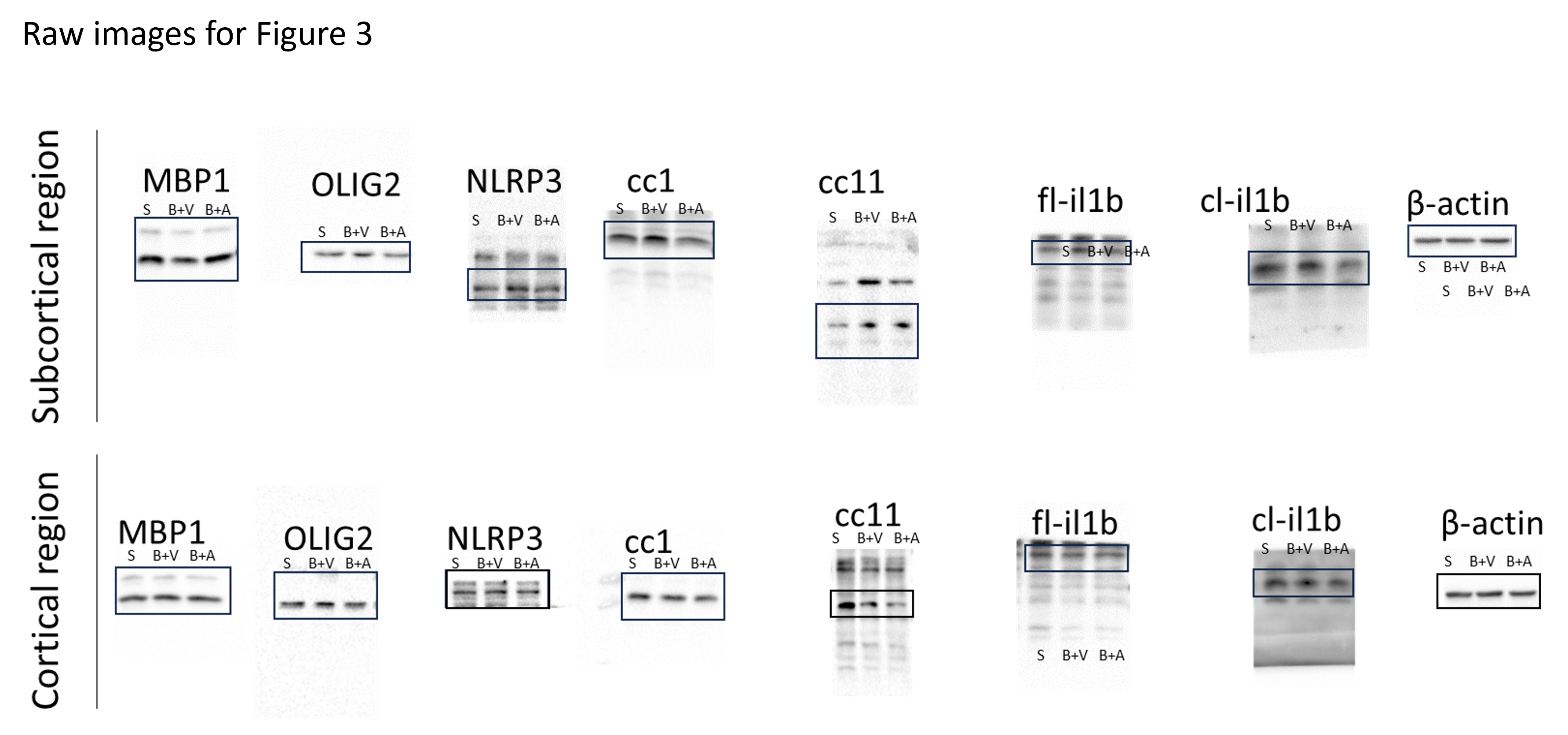

Supplement: S1 Fig — Western blots for Fig 3. (TIF) [file pone.0299703.s001.tif]

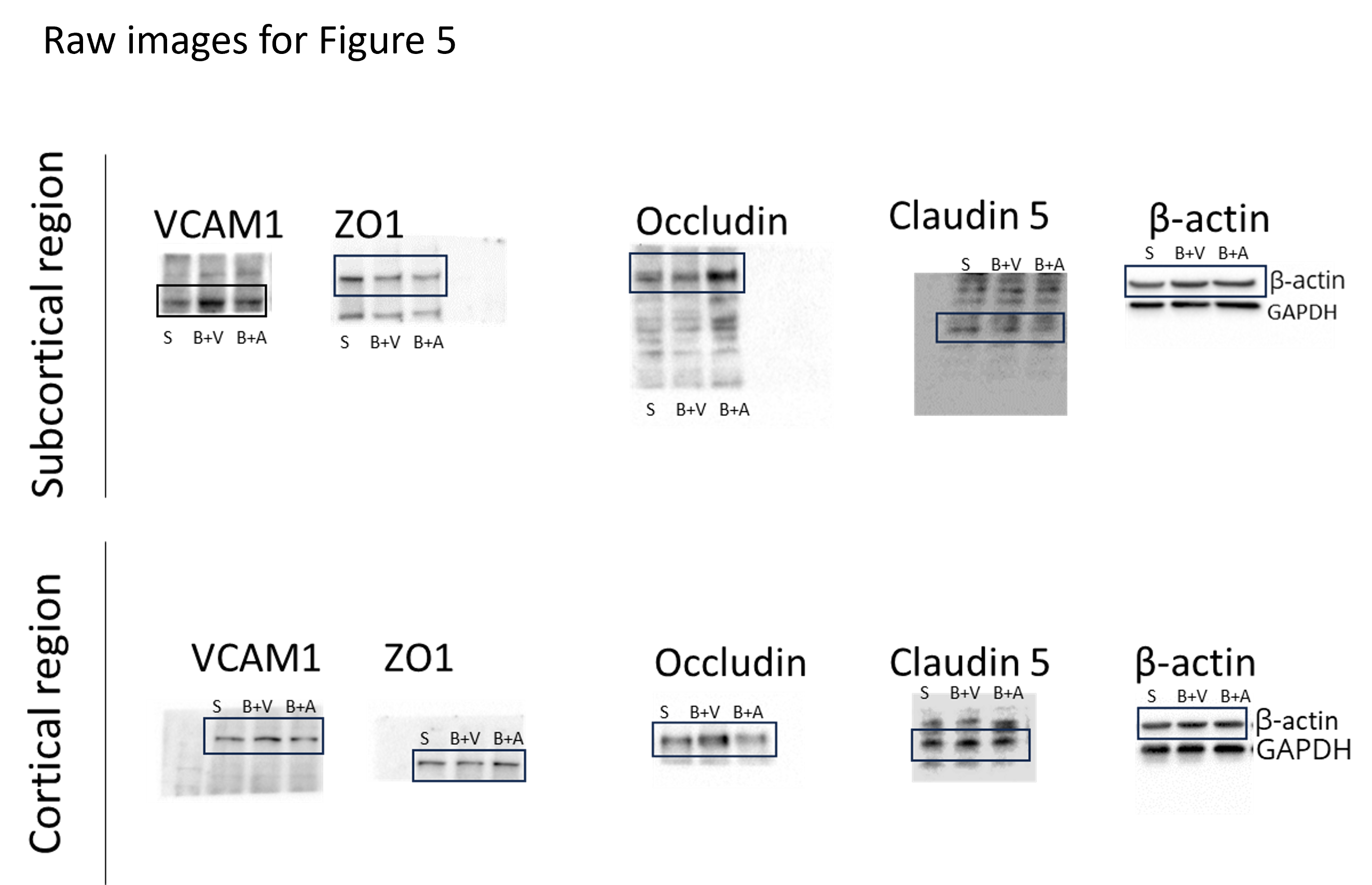

Supplement: S2 Fig — Western blots for Fig 5. (TIF) [file pone.0299703.s002.tif]
